# New insights into the genus *Byzantinia* (Cricetodontinae, Rodentia) from the Late Miocene of Lebanon

**DOI:** 10.7717/peerj.21543

**Published:** 2026-07-22

**Authors:** Raquel López-Antoñanzas, Fabien Knoll, Dany Azar, George Kachacha, Josep Sanjuan, Ebru Albayrak, Pablo Peláez-Campomanes

**Affiliations:** 1 ISEM, Université de Montpellier, CNRS, IRD, Montpellier, France; 2 Departamento de Paleobiología, Museo Nacional de Ciencias Naturales-CSIC, Madrid, Spain; 3 Natural Sciences Department, Faculty of Sciences II, Lebanese University, Fanar, Lebanon; 4 Nanjing Institute of Geology and Paleontology, Chinese Academy of Sciences, Nanjing, China; 5 Departamento de Dinámica de la Tierra y del Océano, Facultad de Ciencias de la Tierra, Universitad de Barcelona, Barcelona, Spain; 6 MTA Şehit Cuma Dağ Natural History Museum, Ankara, Turkey

**Keywords:** Byzantinia, Zahleh 16, Lebanon, Dental morphology, Molar evolution, Miocene

## Abstract

*Byzantinia* is a genus of extinct cricetodontine rodents with a distribution centered in the eastern Mediterranean (Greece, Turkey, and Lebanon) and extending into Romania. It originated during the Middle Miocene in Anatolia and became extinct at the end of the Miocene, its last representatives being *Byzantinia pikermiensis*, *B. uenayae*, and *B. hellenicus*. Several isolated molars attributed to *B. pikermiensis* and *B. hellenicus* have been recovered from a newly discovered fossiliferous locality in Lebanon (Zahleh 16). This material provides new insights into the occlusal morphology of the genus and allows the identification of several evolutionary trends. The co-occurrence of *B. pikermiensis* and *B. hellenicus* suggests an age of 8.9–7.5 Ma for Zahleh 16. Given that the earliest record of *Byzantinia* in Lebanon dates to ca. 10.9–10 Ma, these findings indicate that the presence of the genus in this area persisted for more than 1 million years.

## Introduction

Cricetodontinae is a subfamily of extinct rodents that originated during the Late Oligocene/Early Miocene and includes the genera *Cricetodon*, *Byzantinia*, *Hispanomys*, and *Ruscinomys* (de Bruijn & Ünay, 1996). These animals underwent a major diversification and achieved a wide geographic distribution from their area of origin, Anatolia, to Europe and Asia. During the Middle Miocene, cricetodontines were particularly successful in Europe and Anatolia until their extinction in the Pliocene.

Recent studies have greatly improved the understanding of the phylogenetic relationships within this group, the origin of its main lineages, and its dispersal events (López-Antoñanzas & Peláez-Campomanes, 2022). The early evolution of cricetodontines took place in Anatolia at approximately 25 Ma, when several lineages differentiated before dispersing into Greece, Europe, and Asia (López-Antoñanzas & Peláez-Campomanes, 2022; López-Antoñanzas et al., 2024). During the late Middle and Late Miocene, the evolution of cricetodontines was characterized by two dynamic speciation centres: one in southern and western Europe with the genus *Hispanomys*, and another in Anatolia with the genus *Byzantinia*. *Byzantinia* originated in Anatolia during the late Middle Miocene and became extinct at the end of the Miocene. It represents one of the most characteristic cricetodontine lineages of the eastern Mediterranean region, with occurrences documented in Anatolia, Greece, and more recently in Romania (Badea, Ratoi & Brânzilă, 2023) and Lebanon (López-Antoñanzas et al., 2024; this study). The discovery of *Byzantinia rosamariae* in Lebanon provided the first clear evidence of dispersal of this lineage into Afro-Arabia around 11 Ma (López-Antoñanzas et al., 2024). Despite these recent discoveries, the dental morphology and diversity of *Byzantinia* in the eastern Mediterranean remain incompletely documented, particularly in the Levantine region. In this context, the fossiliferous deposits of Zahleh (Bekaa Valley, Lebanon) provide a rare opportunity to improve our knowledge of this genus in this region. Previous work has identified several micromammal-rich fossiliferous levels within the Zahleh Formation (López-Antoñanzas et al., 2015, 2019, 2024). The oldest of these, layer 3, has yielded the species *Byzantinia rosamariae* (López-Antoñanzas et al., 2024). The present study focuses on new material recovered from a younger level, Zahleh 16, which has also yielded a diverse micromammal assemblage, including two *Byzantinia* species distinct from those recorded in the older fossiliferous level. It provides the first description of fossil rodents from Zahleh 16 and documents their molar morphology and variation.

### Geological settings

**Figure 1:**
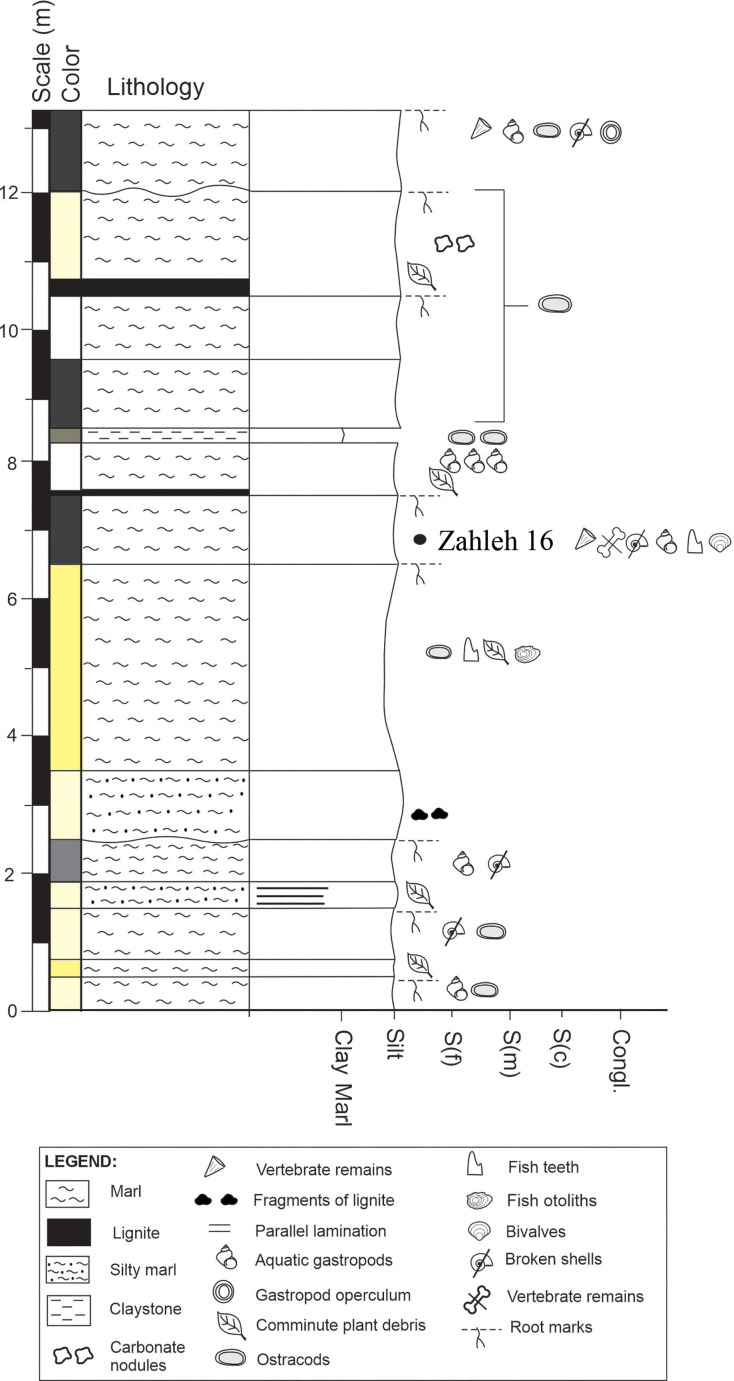
Section 5 in the Zahleh area (Lebanon). Stratigraphic column of the basal part of the Section 5 (Zahleh area, Lebanon) showing the layer (no. 16) from which *Byzantinia pikermiensis and B. hellenicus* have been recorded.

Fieldwork carried out in the summers of 2013 and 2015 in Miocene deposits near the town of Zahleh (Bekaa Valley, Lebanon), led to the discovery of two stratigraphic sections (recently named Section 1 and Section 5 by Khairallah (2019)), which include various fossiliferous layers (López-Antoñanzas et al., 2015, 2019, 2024). These deposits belong to the upper part of the Zahleh Formation, a continental sedimentary sequence composed of clastic materials, marls, limestones, and lignites. *Byzantinia rosamariae* (López-Antoñanzas et al., 2024) was found from the oldest locality, which is located in Section 1 (layer 3 *sensu* López-Antoñanzas et al., 2015; “Zahleh 2” *sensu* Sanjuan et al., 2019). Section 1 is a road cut, the stratigraphic sequence of which has been presented previously (López-Antoñanzas et al., 2015, figure 1). These deposits were initially considered “Pontian” in age (*i.e*., 5.3 Ma, the former Miocene-Pliocene boundary) by Dubertret (1955), Kansou (1961), and Haj-Chahine (1973). However, the study of their micromammal assemblages by López-Antoñanzas et al. (2015, 2019, 2024) indicated a much older age (*circa* 10.5 Ma). The present work focuses on the second stratigraphic section, Section 5, which was also discovered in 2013 near Zahleh. This section became visible thanks to excavation works that provided a fresh outcrop and an opportunity to observe the vertical profile. It comprises 24 m of fossiliferous marls, claystones, and silty marls (Fig. 1). Section 5 is dominated by metric strata of dark organic fossiliferous marls with root marks, alternating with cream-coloured marls and silty intervals, all attributed to palustrine and shallow lake facies. The sedimentary succession contains abundant microfossils, as previously reported (Khairallah, 2019). The basal part of this section, a layer designated Zahleh 16 (~1 m of dark grey marls with root marks), has yielded a variety of micromammals, together with other vertebrate remains such as fishes and crocodiles. Among the micromammals, several molars have been attributed to two species of *Byzantinia*, which together with other rodent remains, indicates that the deposits of Section 5 are younger than those of layer 3 in Section 1.

## Materials and Methods

The teeth described in this work have been compared with those belonging to all known species of *Byzantinia* (see Table S1): *Byzantinia candirensis*, *Byzantinia cariensis*, *Byzantinia eskihisarensis*, *Byzantinia sofcaensis*, *Byzantinia ozansoyi*, *Byzantinia bayraktepensis*, *Byzantinia nikosi*, *Byzantinia rosamariae*, *Byzantinia dardanellensis*, *Byzantinia pikermiensis*, *Byzantinia uenayae*, *Byzantinia hellenicus*. Sarica-Filoreau (2002) described a new species of *Byzantinia* in her PhD thesis, which is still pending formal publication. This species is included in the present work as “*Byzantinia* sp. from Direcik I” (Turkey). First, second, and third lower molars are designated as m1, m2, and m3 and first, second, and third upper molars as M1, M2, and M3, respectively.

**Figure 2:**
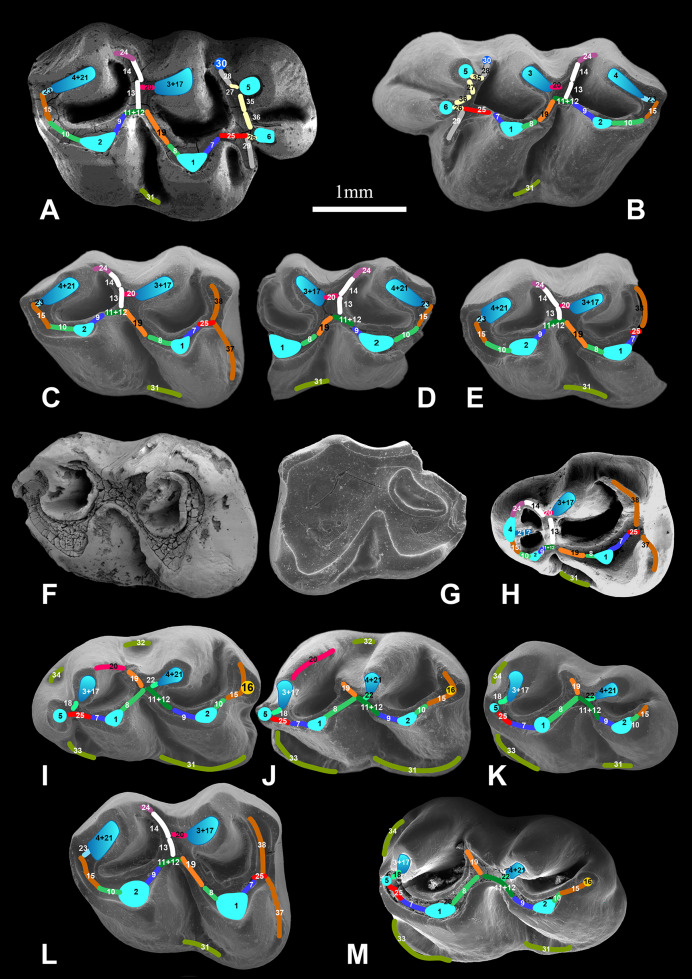
Dental terminology used in this study, marked on the *Byzantinia* material examined here: *Byzantinia pikermiensis* (A)–(K) and to *B. hellenicus* (L)–(M). Catalogue numbers are specified in the legend of Fig. 3. Scale bar represents 1 mm.

Previous phylogenetic studies that we have conducted have highlighted challenges in categorizing dental structures in rodents (*e.g*., López-Antoñanzas & Peláez-Campomanes, 2022; López-Antoñanzas et al., 2024; Dirnberger, Peláez-Campomanes & López-Antoñanzas, 2024; Dirnberger et al., 2026). Recent work by Barbière et al. (2019), which proposes the existence of hybrid structures in the dental morphology of sigmodontine rodents, has led us to recognize the advantages of separating structures, generally considered as single for most authors and therefore named as one structure, into distinct components in order to better categorize different species. Accordingly, in this work we identify and name crests that form part of hybrid structures which were previously treated as a single feature, but which we consider important to distinguish. Thus, we apply here a dental terminology currently under development [R. López-Antoñanzas & P. Peláez-campomanes, 2026, in review] which complements existing terminologies for cricetodontine rodents (Mein & Freudenthal, 1971b; López-Antoñanzas et al., 2010; López-Guerrero, García-Paredes & Álvarez-Sierra, 2013; López-Guerrero et al., 2014). This approach is based on a more detailed characterization of dental structures, particularly in cases where complex or hybrid features are involved. The terminology is applied here for descriptive purposes to the genus *Byzantinia*, a highly specialized taxon, and allows a more precise description of its dental morphology (Fig. 2, Table 1).

**Table 1:** Terminology of the different structures identified in the genus *Byzantinia*. Numbers correspond to the structures shown in Fig. 2.

	Upper molars	Lower molars
1	Protocone	Protoconid
2	Hypocone	Hypoconid
3	Paracone	Metaconid
4	Metacone	Entoconid
5	Labial anterocone	Linguan anteroconid
6	Lingual anterocone	Labial anteroconid
7	Anterior arm of the protocone	Anterior arm of the protocone
8	Posterior arm of the protocone	Posterior arm of the protocone
9	Anterior arm of the hypocone	Anterior arm of the hypoconid
10	Posterior arm of the hypocone	Posterior arm of the hypoconid
11	Anterior longitudinal crest	Anterior longitudinal crestid
12	Posterior longitudinal crest	Posterior longitudinal crestid
13	Mesoloph lingual part	Mesolophid labial part
14	Mesoloph labial part	Mesolophid lingual part
15	Labial posteroloph	Lingual posterolophid
16	Hypoconule	Hypoconulid
17	Paraloph	Metalophid
18	Protolophule I	Metalophulid I
19	Protolophule II (lingual part)	Metalophulid II
20	Posterior paracone spur	Posterior metaconid ridge
21	Metaloph	Entolophid
22	Hypolophule I	Hypolophulid
23	Hypolophule II	
24	Anterior metacone spur	
25	Anterolophule	Anterolophulid
26	Posterior spur of lingual anterocone	
27	Posterior spur of labial anterocone	
28	Parastyle lingual spur	
29	Lingual spur of the anterolophule	
30	Parastyle	
31	Lingual cingulum	Labilal cingulid
32	Mesocingulum	Mesocingulid
33	Anterolingual cingulum	Anterolabial cingulid
34	Anterolabial cingulum	Anterolingual cingulid
35	Lingual spur of labial anterocone	
36	Labial spur of lingual anterocone	
37	Lingual anteroloph	Labial anterolophid
38	Labial anteroloph	Lingual anterolophid

Measurements of the occlusal surface of the teeth (maximum length and maximum width) have been taken following the method of Van de Weerd (1976) for all dental elements but the M2s, for which the maximum length has been taken parallel to the labial side of the tooth. Measurements were obtained with a Nikon digital counter CM-6S measuring device.

Fieldwork was conducted with the appropriate authorization to access the study areas. According to local regulations, no specific fieldwork permit was required for this work. The field activities were carried out in full compliance with applicable local laws and institutional norms. Given these circumstances, no additional formal permit documentation exists.

## Results

SYSTEMATICS

Order RODENTIA Bowdich, 1821

Superfamily MUROIDEA Illiger, 1811

Subfamily CRICETODONTINAE Stehlin et Schaub, 1951

Tribe CRICETODONTINI Simpson, 1945

Genera *BYZANTINIA* de Bruijn, 1976

Type species: *Byzantinia pikermiensis* de Bruijn, 1976

***Byzantinia pikermiensis***

(Figs. 3A–3K)

**Figure 3:**
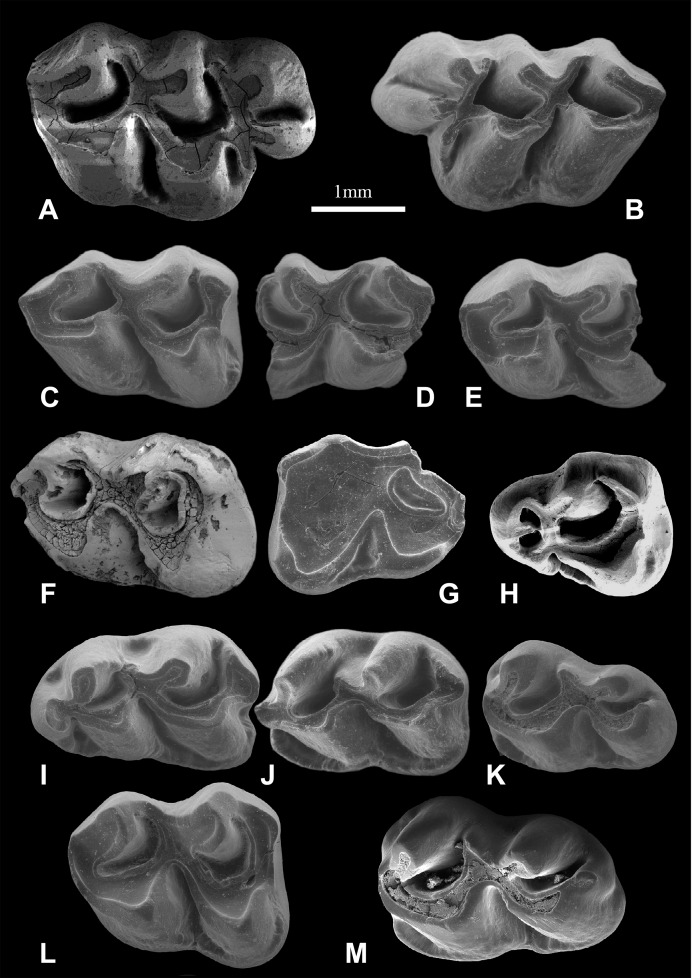
*Byzantinia pikermiensis* (A)–(K) and *Byzantinia hellenicus* (L), (M). (A) Left M1 (LUNHN-F-51153/Zahleh-16-72); (B) right M1 (LUNHN-F-51153/Zahleh-16-01); (C) right M2 (LUNHN-F-51153/Zahleh-16-03); (D) left M2 (LUNHN-F-51153/Zahleh-16-05); (E) right M2 (LUNHN-F-51153/Zahleh-16-04); (F) right M2 (LUNHN-F-51153/Zahleh-16-73); (G) left M2 (LUNHN-F-51153/Zahleh-16-06); (H) right M3 (LUNHN-F-51153/Zahleh-16-74); (I) left m1 (LUNHN-F-51153/Zahleh-16-08); (J) left m2 (LUNHN-F-51153/Zahleh-16-10); (K) left m3 (LUNHN-F-51153/Zahleh-16-11); (L) right M2 (LUNHN-F-51153/Zahleh-16-02); (M) left m3 (LUNHN-F-51153/Zahleh-16-71). Scale bar represents 1 mm.

*Material*. Left M1 (LUNHN-F-51153/Zahleh-16-72, Fig. 3A), right M1 (LUNHN-F-51153/Zahleh-16-01, Fig. 3B), right M2 (LUNHN-F-51153/Zahleh-16-03, Fig. 3C), anteriorly broken left M2 (LUNHN-F-51153/Zahleh-16-05, Fig. 3D), anteriorly broken right M2 (LUNHN-F-51153/Zahleh-16-4, Fig. 3E), right M2 (LUNHN-F-51153/Zahleh-16-2, Fig. 3F), left M2 (LUNHN-F-51153/Zahleh-16-06, Fig. 3G), right M3 (LUNHN-F-51153/Zahleh-16-74, Fig. 3H), left m1 (LUNHN-F-51153/Zahleh-16-08, Fig. 3I), left m2 (LUNHN-F-51153/Zahleh-16-10, Fig. 3J), left m3 (LUNHN-F-51153/Zahleh-16-11, Fig. 3K). All specimens are housed at the Natural History Museum of the Lebanese University (LUNHN) Fanar under the care of Dr. Dany Azar.

*Locality*. Zahleh 16, Wadi Al Aarayech, Zahleh, Lebanon. All specimens originate from a dark grey marls layer with root marks.

*Age*. Late Miocene, Late Tortonian (~8.9–7.6 Ma)

*Emended diagnosis*. Medium-sized species of *Byzantinia* with moderate hypsodont teeth. Upper molars having a well-developed lingual spur of the anterolophule and lacking a funnel structure. Third upper molars elongated and posteriorly strongly reduced, with very small and lingually displaced metacone. Lower molars showing an anterior metalophulid (I), lacking mesolophid, having a short posterior metalophulid (II) directed towards the metaconid and a rather strong anterolabial cingulid near the base of the tooth, particularly on m2; m3 being very elongated with a narrow posterior part, and having a short but well-developed metalophulid II. The hypoconulid decreases in size along the tooth row, being larger on the m1, smaller on the m2 and absent on the m3.

*Measurements* (see Table 2)

**Table 2:** Measurements. Length and width measurements (mm) of the lower and upper molars of *Byzantinia pikermiensis* and *Byzantinia hellenicus* from Zahleh 16 (Lebanon).

Species	Dental element	Catalogue number	Length	Width
*Byzantinia pikermiensis*	M1	ZAHLE16-1	3.39	2.10
*Byzantinia pikermiensis*	M2	ZAHLE16-3	2.57	1.95
*Byzantinia pikermiensis*	M2	ZAHLE16-4		1.91
*Byzantinia pikermiensis*	M2	ZAHLE16-5		
*Byzantinia pikermiensis*	M2	ZAHLE16-6		
*Byzantinia pikermiensis*	M2	ZAHLE16-7		
*Byzantinia pikermiensis*	m1	ZAHLE16-8	2.67	1.74
*Byzantinia pikermiensis*	m1	ZAHLE16-9		
*Byzantinia pikermiensis*	m2	ZAHLE16-10	2.58	1.75
*Byzantinia pikermiensis*	m3	ZAHLE16-11	2.31	1.50
*Byzantinia pikermiensis*	M1	ZAHLE16-72	3.19	2.16
*Byzantinia pikermiensis*	M2	ZAHLE16-73	2.54	1.88
*Byzantinia pikermiensis*	M3	ZAHLE16-74	2.02	1.77
*Byzantinia hellenicus*	M2	ZAHLE16-2	2.49	2.17
*Byzantinia hellenicus*	m3	ZAHLE16-71	2.64	1.83

DESCRIPTION

*Upper molars*

M1—

The anterocone is bilobed, bearing two sub-equal cusps (6 + 5) separated by a wide and deep anteromedial groove. These cusps are connected posteriorly by a relatively long, nearly transverse crest, interpreted here as the result of the junction between the lingual spur of the labial anterocone (35) and the labial spur of the lingual anterocone (36). Both the labial and lingual anterocones additionally present a posterior spur each (27 and 26, respectively). In one of the two specimens (Figs. 2, 3A), a parastyle (30) is present, from which a small crest (28) extends anterolingually to join the posterior spur of the labial anterocone (27). In the second specimen (Figs. 2, 3B), the parastyle (30), if present originally, can no longer be distinguished from crest 28 due to occlusal wear. The anterolophule (25) is well developed but difficult to differentiate from the posterior spur of the lingual anterocone (26), with which it forms a continuous crest. In the less worn specimen (Figs. 2, 3A), a slight constriction or interruption is visible, which we interpret as the boundary between the distal end of the anterolophule and the beginning of the anterior arm of the protocone (7), allowing these crests to be clearly delimited. The forward paracone spur is absent, resulting in an incomplete anterior ectoloph. There is no anteroloph. The paracone (3) appears strongly elongated and posteriorly directed owing to its fusion with the paraloph (17). This latter crest connects with the posterior paracone spur (20), although this structure (20) is short and links to what we interpret as the lingual and labial portions of the mesoloph (13 + 14). The labial portion of the mesoloph (14) joins what we interpret as an anterior metacone spur (24), giving the impression of a single, long structure bending towards the metacone.

The anterior metacone spur (24) is present at mid-crown height between the metaconid and the entoconid. We interpret the mesoloph as being composed of two distinct ridges (13 + 14) and consider the labial part of the posterior protoloph (or protoloph II) to be the structure lost in this derived species of *Byzantinia* (see discussion below). The loss of the labial part of the posterior protoloph results in the absence of the funnel structure (*sensu* Ünay, 1980) that characterizes certain taxa of *Byzantinia*. What remains in these derived species of *Byzantinia* is the lingual portion of this protoloph II (ridge 19).

The anterior (7) and posterior (8) arms of the protocone are relatively short, as evidenced by the interruption or constriction at the level of their connection to the anterolophule (25) and the lingual part of the posterior protoloph (19), respectively, as observed in the less worn specimen (Figs. 2, 3A).

A short posterior arm of the protocone (8) connects to the lingual part of the posterior protoloph or protolophule II (19). There is a clear interruption between the two structures, making it possible to distinguish where one ends and the other begins. Crest 19 connects to the longitudinal crest (11–12). The longitudinal crest is short, compared to the situation in primitive species of Cricetodontinae, such as *Cricetodon verstegi*. In primitive Cricetodontinae, this crest is constituted by the combination of two distinctive ridges (11 + 12) that run from the lingual part of the posterior protoloph (19) and the anterior arm of the hypocone (9), respectively, towards the lingual part of the mesoloph (13). In this derived species, we interpret that one of the two components of this structure may have been lost (probably its anterior part or ridge 11).

The anterior arm of the hypocone (9) descends and tapers posterior to the longitudinal crest (12), from which it remains separated by a distinct interruption. The metaloph (21) and metacone (4) are fused, as are the paracone and paraloph (3 + 17), forming an elongated structure (21 + 4) oriented parallel to the latter complex. The labial posteroloph (15) does not extend beyond the point where it meets the metaloph or the posterior hypolophule (23). The sinus is narrow and deep, slightly backwards, and closed lingually by a well-defined lingual cingulum (31). The teeth are four-rooted.

M2—

These teeth are trapezoidal in outline in occlusal view, with a narrower posterior part. The labial anteroloph (38) is thick and, in the most worn specimens, may reach the anterior side of the paracone (3) at mid-height. The lingual anteroloph (37) is longer, slenderer and extends to the base of the protocone (1). The anterolophule (25) is short and connects the anterior arm of the protocone (7) to both anterolophs. As in M1, the paracone (3) is elongated and fused with the paraloph (17), forming a posteriorly directed structure (3 + 17) that connects to the backward paracone spur (20) and the mesoloph (13 + 14). As in M1, the lack of funnel is due to the loss of the labial part of the protolophule II; the remaining structure being interpreted as the mesoloph (13 + 14). The longitudinal crest is relatively short and, as on M1, probably completely or largely represented by its posterior component (12). As we lack unworn M2 specimens, we are unable to confirm the interruption in this tooth between the posterior arm of the protocone (8) and the lingual part of the posterior protoloph (protolophule II, crest 19) that is observed on unworn M1 (Fig. 2A). Nevertheless, the strong morphological similarity between M2 and M1 allows us to infer that this apparently single crest is actually composed of two separate structures. As observed on the M1, the short anterior arm of the hypocone (9) descends and narrows opposite the longitudinal crest from, which it is separated by a distinct interruption. The metaloph (21) and metacone (4) are merged in an elongated, strongly posteriorly inclined structure that is similar and parallel to the paracone-paraloph (3 + 17).

The labial posteroloph (15) is relatively short and terminates at its junction with the metaloph or the posterior hypolophule (23), which may be indistinguishable from one another. The sinus is narrow, deep, runs slightly posteriorly and is closed by a lingual cingulum (31).

M3—

This tooth is oval in occlusal view. Its posterior part is strongly reduced with the paracone (3) and protocone (1) taking up more than 2/3 of the occlusal surface. The hanging lingual anteroloph (37) is less developed than the labial one and runs from the anterolophule (25) to almost the base of the protocone. The labial anteroloph (38) is high and thick and its labial end connects to the anterior side of the paracone (3) at about half its total height. The posteriorly directed paracone is elongated due to its fusion with the paraloph (17). The protoloph is lacking. As is the case in the other upper molars. A short posterior paracone spur (20) arises from the paracone + paraloph and connects to the mesoloph (interpreted as 13 + 14). The mesoloph joins the anterior metacone spur (24), closing a posterolabial sinus. The longitudinal crest (11 + 12) is short and connects with both the posterior protoloph (19) and the very short anterior arm of the hypocone (9). The metacone (4) is medially positioned, aligned with the longitudinal axis of the tooth. The labial portion of the anterior metaloph (21) arises from it in form of a longitudinal spur. It extends anteriorly along the longitudinal axis of the tooth toward what we interpret as the lingual part of the mesoloph (13), without reaching it. The protocone is labio-lingually compressed. A lingual cingulum (31) is well developed and nearly closes the posteriorly directed sinus. The roots of this tooth are not preserved.

*Lower molars*

The lower molars are subequal in length, with the m3 being only slightly shorter than the m1 and m2.

m1—

The occlusal outline of this tooth is triangular, longer than wide, with its anterior part rounded and narrower than the posterior one. The anteroconid (5) is a well-developed, round cuspid. The lingual anterolophid is absent, but there is a weak anterolingual cingulid (34). The anterolabial cingulid (33) is rather small and runs down to connect to the anterior side of the protoconid enclosing the protosinusid. As interpreted for the upper molars, both the metaconid (3) and entoconid (4) may have merged with the metalophid (17) and the entolophid (21), respectively, forming two anteriorly directed and elongated structures. The metaconid-metalophid (3 + 17) complex connects to the anterolophulid (25) through metalophulid I (18). The anterolophulid (25) is well developed and joins the anteroconid by mean of the rather short anterior arm of the protoconid (7). The posterior arm of the protoconid (8) is well developed and connects to the longitudinal crest (11 + 12). The latter appears to have lost or shortened its anterior portion (11), whereas its posterior segment (12) is steeply inclined and represents a continuation of the anterior arm of the hypoconid (9). From the point where the longitudinal crest and the posterior arm of the protoconid join, a relatively short, lingual and anteriorly directed crest arises (19). The examination of numerous specimens of primitive cricetodontine species belonging among others to the genera *Cricetodon* and *Hispanomys*, as well as the observation of a wide range of species of *Byzantinia*, leads us to interpret the crest 19 as the labial part of the posterior metalophulid, or metalophulid II. In previous studies, this structure has also been referred to as the mesolophid, reflecting differences in interpretation among authors. In any case, crest 19 connects with an irregular posterior metaconid ridge (20). From the entoconid–entolophid complex (4 + 21) arises the lingual hypolophulid (22), which connects to the longitudinal crest. The posterior arm of the hypoconid is short and continues into the lingual posterolophid (15), which is high and bears a hypoconulid (16). A labial cingulid (31), running from the base of the protoconid, encloses the sinusid. This latter structure is deep, rather narrow and directed forwards. A distinct lingual mesocingulid (32) is present at the base of the crown between the metaconid and the entoconid. The roots of this tooth are not preserved.

m2—

This tooth is rectangular in occlusal outline, with an anterior portion that is less rounded than in m1, and bears an anterior cusp that is still recognizable as the anteroconid (5). Overall, the morphology closely resembles that of m1; however, several differences are evident. Notably, m2 lacks an anterolingual cingulid (34) and possesses a anterolabial cingulid (33) that is considerably stronger and longer than that of m1. This latter hangs near the base of the tooth, extending from the anterior portion of the tooth below the anteroconid to the lingual base of the protoconid, enclosing a protosinusid. Other distinguishing features include the posterior metaconid ridge (20), which is longer on m2 and connects to the mesocingulid (32) near the base of the crown, rather than joining metalophulid II (19) as is the case in m1. Finally, although m2 exhibits a distinct hypoconulid, it is less developed than that observed on m1. The roots of this tooth are not preserved.

m3—

The morphology of this tooth is quite similar to of m2. The main difference is that m3 has a reduced and rounded posterior part. The anteroconid (5) is no longer distinct as an individual cusp, and the posterior metaconid ridge (20) and the mesocingulid (32) are absent. The anterolabial cingulid (33) is less developed than in m2, yet still developed enough to enclose the protosinusid. As observed on m2, metalophulid II (19) is short and ends free, so the mesosinusid remains open. The posterior arm of the hypoconid (10) and the lingual posterolophid (15) are very short and indistinguishable from one another, and the hypoconulid (16) is lacking. The sinusid is closed by a distinct labial cingulid (31). The roots of this tooth are not preserved.

**Figure 4:**
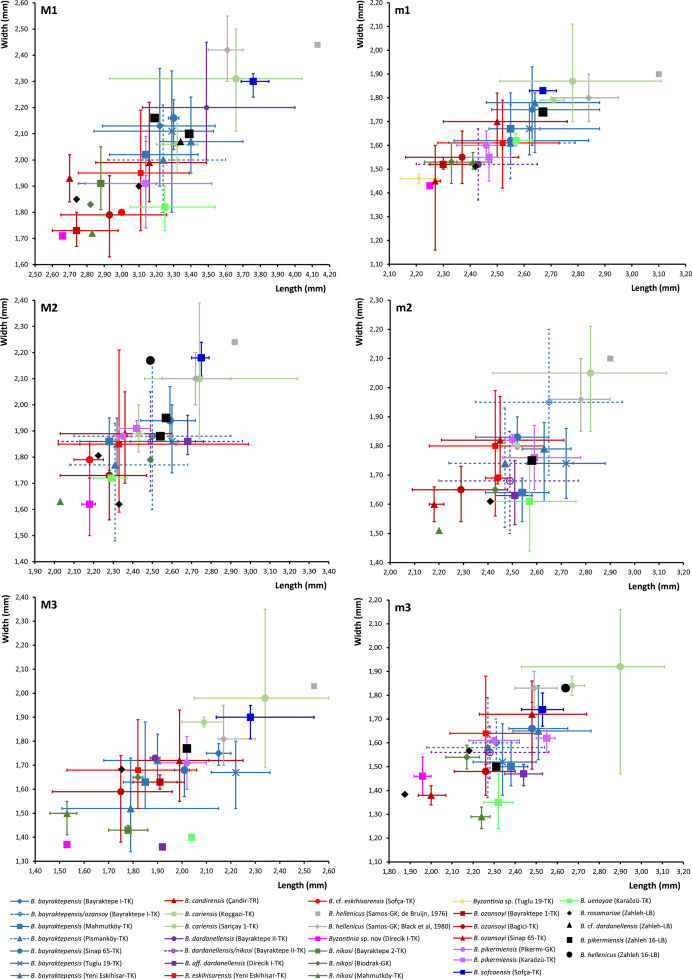
Length/width scatter diagrams of the upper and lower molars of all the species of *Byzantinia* from various localities. The black square indicates the size of *Byzantinia pikermiensis* and the black circle that of *Byzantinia hellenicus* from the Lebanese locality Zahleh 16 studied here.

*Comparison*. The small teeth of *Byzantinia* from Zahleh 16 are larger than those of *Byzantinia rosamariae*, *B. nikosi*, *B. ozansoyi*, and *Byzantinia* sp. from Direcik I (Fig. 4). The Lebanese taxon differs from *B. eskihisarensis*, *B. candirensis*, *B. cariensis*, *B. ozansoyi*, *B. bayraktepensis*, *Byzantinia* sp. from Direcik I, and *B. dardanellensis* in having a lingual spur of the anterolophule on M1 (ridge 29). Among the genus, this structure is otherwise only known in *B. sofcaensis*, *B. nikosi*, *B. rosamariae*, *B. pikermiensis*, *B. hellenicus*, and *B. uenayae*. Moreover, the Lebanese taxon differs from *B. candirensis*, *B. cariensis*, *B. eskihisarensis*, *B. sofcaensis*, *B. ozansoyi*, *B. nikosi*, *Byzantinia* sp. from Direcik I, and *B. rosamariae* (Figs. 2, 3) in lacking a funnel structure (*sensu* Ünay, 1980) on M1 and M2. In addition, the material from Zahleh 16, as well as *B. pikermiensis*, differs from all other species of the genus but *B. sofcaensis*, *B. dardanellensis*, and *B. hellenicus* in having the two cusps of the anterocone widely separated and connected by a well-developed, relatively transverse crest. Contrary to the condition observed in *C. pasalarensis*, *B. sofcaensis*, *B. ozansoyi*, *B. bayraktepensis*, *B. rosamariae*, and *Byzantinia* sp. from Direcik I, M2 of *Byzantinia* from Zahleh 16 appears elongated as a result of the reduction of its posterior part, a condition also observed in *B. pikermiensis*. Furthermore, the morphology of M3 of the Lebanese species is characterized by a markedly reduced posterior part, with a very small and lingually shifted metacone bearing a longitudinal spur (anterior metaloph) that extends anteriorly/along the longitudinal axis toward the mesoloph. This condition closely resembles that observed in the M3 of *B. pikermiensis* from Çorakyerler (RLA and PPC personal observations but see also Ünay, de Bruijn & Suata-Alpaslan, 2006).

Interestingly, all teeth from Zahleh 16 except m1 fall within the size range of *B. pikermiensis* (Fig. 4). Although the single Lebanese m1 recovered is unexpectedly larger than those of that species, the strong morphological similarity and the size overlap observed in the remaining teeth with *B. pikermiensis* support the attribution of the small-sized material from Zahleh 16 to this species.

***Byzantinia hellenicus***

*Material*. Right M2 (LUNHN-F-51153/Zahleh-16-02, Fig. 3L), left m3 (LUNHN-F-51153/Zahleh-16-71, Fig. 3M). The specimens are stored at the Natural History Museum of the Lebanese University (Faculty of Sciences II) Fanar under the care of Dr. Dany Azar.

*Locality*. Zahleh 16, Wadi Al Aarayech, Zahleh, Lebanon. The two specimens come from a dark grey marls layer with root marks.

*Age*. Late Miocene, Late Tortonian (~8.9–7.6 Ma)

*Measurements* (see Table 2)

DESCRIPTION

M2—

A single, poorly preserved M2 has been recovered, the morphology of which is nearly identical to that observed in M2 from the same level assigned to *Byzantinia pikermiensis* but its width is noticeably larger. The tooth is trapezoidal in occlusal view, with a narrower posterior part, and shows the same general pattern of crest connections and reductions observed in that species (for a detailed description see *B. pikermiensis*).

m3—

This tooth closely resembles those of *B. pikermiensis* in morphology but clearly exceeds them in size (Fig. 4). It differs slightly morphologically in having a less reduced posterior part, with a distinct hypoconulid (16), which is absent in *B. pikermiensis*.

**Comparison.** The dental morphology of the two large *Byzantinia* teeth from Lebanon is nearly identical to that of *B. pikermiensis* and *B. hellenicus*. However, they clearly exceed *B. pikermiensis* in size. Together with *B. hellenicus*, *B. cariensis*, and *B. sofcaensis*, these specimens rank among the largest representatives of the genus known to date. The Lebanese specimens are, however, morphologically more derived than those of *B. cariensis* and *B. sofcaensis*, for instance in lacking a funnel on the upper molars. Although the m3 from Zahleh 16 exceeds the size range reported by Black, Krishtalka & Solounias (1980) for *B. hellenicus*, its morphology closely matches that species. The measurements reported by Black, Krishtalka & Solounias (1980) are based on complete mandibles rather than isolated teeth, and the limited sample size prevents a full assessment of size variability. We therefore consider the attribution of this specimen to *B. hellenicus* justified.

## Discussion

The cricetodontine *Byzantinia* originated in Anatolia during the late Middle Miocene and became extinct by the end of the Miocene. The genus was established by de Bruijn (1976) to distinguish the Turkish and Greek Middle and Late Miocene associations of cricetodontines (*Byzantinia*) from the southwestern European ones (*Hispanomys*). These two genera, in fact, represent distinct lineages that evolved independently since the Middle Miocene (López-Antoñanzas & Peláez-Campomanes, 2022; López-Antoñanzas et al., 2024). Despite displaying similar evolutionary trends, these two groups differ markedly in their dental morphology, particularly in the structure of the third upper molars and in the number of roots of the M1.

Twelve species of this genus are currently recognized: *Byzantinia candirensis* (Tobien, 1978), *B. sofcaensis* (Tobien, 1978), *B. cariensis* (Sen & Ünay, 1979), *B. eskihisarensis* (Tobien, 1978), *B. ozansoyi* Ünay, 1980, *B. bayraktepensis* Ünay, 1980, *B. rosamariae* López-Antoñanzas et al., 2024, *B. nikosi* de Bruijn, 1976, *B. dardanellensis* Ünay, 1980, *B. pikermiensis* de Bruijn, 1976, *B. uenayae* Rummel, 1998, and *B. hellenicus* (Freudenthal, 1970). Moreover, an additional, yet unpublished species, was named by Sarica-Filoreau (2002), and is herein referred to as “*Byzantinia* sp. from Direcik I”. *Cricetodon candirensis* (Tobien, 1978) and *C. cariensis* (Sen & Ünay, 1979) have recently been reassigned to this genus (López-Antoñanzas et al., 2024). According to this latter study, *Cricetodon pasalarensis* (Tobien, 1978) may also be an early representative of *Byzantinia* (but see also López-Antoñanzas & Peláez-Campomanes, 2022). Sen (2016) considered *Ruscinomys orientalis* (Lungu, 1981) from Buzhor-1 (Moldova) to be a possible synonym of *Byzantinia bayraktepensis*. This generic re-allocation would extend the known geographical range of *Byzantinia* to Moldova. However, he remained cautious and emphasized that the material described by Lungu (1981) required new measurements before any taxonomic conclusion could be drawn. We consider that the presence of five roots on M1 together with the morphology of M3 supports Lungu’s original interpretation, suggesting that this form may instead belong to the *Hispanomys*–*Ruscinomys* lineage. Similarly, the *Byzantinia* has been reported from several Late Miocene sites in Romania (Badea, Ratoi & Brânzilă, 2023). Although the material is scarce, the occurrence of three roots on M1 (D. Badea, personal communications, 2025) supports attribution to this genus. Together with the record of *B. rosamariae* in Lebanon (López-Antoñanzas et al., 2024), these findings confirm the presence of *Byzantinia* in Anatolia, Greece, Romania, and Lebanon.

The evolution of the dental pattern of *Byzantinia* can be inferred despite the limited material available for most species. One of the most significant dental characters is the presence of a “funnel” (*sensu* Ünay, 1980), which occurs in some species of *Byzantinia* but is absent in the most derived ones (*B. bayraktepensis*, *B. dardanellensis*, *B. nikosi*, *B. pikermiensis*, *B. uenayae*, *B. hellenicus*; see Ünay, 1980; de Bruijn, 1976; Rummel, 1998; Freudenthal, 1970). The funnel appears to result from the connection between the labial part of protolophule II, the longitudinal crest (11 + 12), the backward paracone spur (20), and the mesoloph (13 + 14). In some taxa such as *B. candirensis*, *B. sofcaensis*, *B. eskihisarensis*, *Byzantinia* sp. from Direcik I, and *B. rosamariae* (Sen & Ünay, 1979; Tobien, 1978; Sarica-Filoreau, 2002; López-Antoñanzas et al., 2024), this funnel may be interrupted lingually, when the anterior portion of the longitudinal crest (11) partially disappears and becomes disconnected from the posterior protolophule. We suggest that in the most derived species of *Byzantinia*, the absence of funnel is due to the disappearance of the labial part of the protolophule, leaving only the mesoloph (13 + 14) as the structure connecting to the posterior paracone spur (20). The widespread interpretation of the mesoloph as a single crest in cricetid-like rodents (*e.g.,* de Bruijn, 1976; Mein & Freudenthal, 1971a; Ünay, 1980; Çinar Durgut & Ünay, 2016; Hír, 2017) is probably misleading. We consider that this ridge actually consists of two distinct elements: a lingual one (13), originating from the longitudinal crest and extending labially, and a labial one (14), arising from the mesostyle (if present) or from the labial side of the tooth and extending lingually (13). The interpretation of the mesoloph as a structure composed of two crests has already been advanced for other groups of early rodents (Flynn, Jacobs & Cheema, 1986; Marivaux, Vianey-Liaud & Jaeger, 2004; Vianey-Liaud & Marivaux, 2017).

The most primitive species of *Byzantinia* have the lingual part (19) of the posterior protolophule shorter than the labial one. This character can be observed in *B. candirensis*, *B. cariensis*, *B. eskihisarensis*, *B. sofcaensis*, *B. ozansoyi, Byzantinia* sp. from Direcik I, and *B. rosamariae* (Tobien, 1978; Ünay, 1980; Sarica-Filoreau, 2002; López-Antoñanzas et al., 2024). During the evolution of *Byzantinia*, the lingual part of the protolophule (19) lengthened, whereas the labial one shortened, becoming hardly distinguishable from the paracone-paraloph complex (3 + 17) or disappearing entirely in derived species of the genus such as *B. bayraktepensis*, *B. dardanellensis*, *B. nikosi*, *B. pikermiensis*, *B. uenayae*, and *B. hellenicus* (Ünay, 1980; de Bruijn, 1976; Freudenthal, 1970).

Some changes in the morphology of the anterocone (5–6) are also evident in M1 of *Byzantinia*. In the most derived species of this genus, the two anterocones are distinctly separated from each other and connected by a relatively long transverse ridge (35 + 36), a clear example of which can be observed in the material of *B. pikermiensis* described here (Figs. 2, 3). However, it is important to note that a marked separation between the two anterocones is not exclusive to the more derived species; some comparatively primitive forms, such as *Byzantinia sofcaensis* (Tobien, 1978), also exhibit clearly separated anterocones. This suggests that a pronounced separation of the anterocones evolved independently more than once within the group, representing a case of paralell evolution of a dental trait, a phenomenon not uncommon in the modification of the occlusal pattern of rodent species over time.

Similarly, the lingual spur of the anterolophule (29) seems to have been acquired independently more than once during the course of evolution. This structure is observed in some of the most plesiomorphic species of *Byzantinia*, such as *B. sofcaensis* or *B. candirensis* (Tobien, 1978), and also in even more primitive, closely related species (*e.g*., *C. pasalarensis*, Tobien, 1978). However, this structure is also present in the most derived ones such as *B. nikosi*, *B. rosamariae*, *B. pikermiensis*, *B. hellenicus*, and *B. uenayae* (de Bruijn, 1976; Freudenthal, 1970; López-Antoñanzas et al., 2024; Rummel, 1998).

Another character shared by several plesiomorphic taxa such as *B. cariensis*, *B. candirensis*, *B. sofcaensis*, and *C. pasalarensis* (Tobien, 1978) is the position of the posterior arm of the protocone (8), which is placed more anteriorly on the upper molars than in more derived forms. As a result, particularly on M2, this structure can originate from the labial side of the protocone near its midline, giving the misleading impression that the protocone bears a longitudinal spur. This tendency for the posterior arm of the protocone to be positioned somewhat anteriorly is also observed in other cricetodontine genera, such as *Cricetodon* (*e.g*., *C. bolliegeri*, *C. jotae*, *C. hungaricus*; see Rummel, 1995; Mein & Freudenthal, 1971b; López-Guerrero et al., 2014; Hír, 2017) and *Hispanomys* (*e.g*., *H. aragonensis*, *H. lavocati*; see López-Guerrero et al., 2019), but not in more derived species such as *H. adroveri*, which lacks a longitudinal crest, nor in taxa belonging to the genus *Ruscinomys* (Adrover, 1986).

In *Byzantinia*, M3 evolves from a morphology that retains the labial part of the protolophule and exhibits a slightly reduced posterior region, in which the metacone is not reduced and only slightly lingually displaced and the metaloph (21), the mesoloph (13 + 14), and even a short posteroloph (15) remain as identifiable structures (*e.g*., *B. cariensis*, *B. candirensis*, or *B. eskihisarensis* and see Fig. 2H). Taxa closely related to, and even more basal than, the previously mentioned species, such as *Cricetodon pasalarensis* (López-Antoñanzas & Peláez-Campomanes, 2022; López-Antoñanzas et al., 2024), retain a fully developed M3, with well-defined protoloph, anterior metaloph, and posteroloph.

The reduction of the posterior part of M3 in *Byzantinia* leads to a progressive diminution and lingual displacement of the metacone. Throughout the evolutionary history of the genus, the metacone became increasingly reduced until it ultimately merged with the hypocone and the posteroloph to form a single crest, as observed in *Byzantinia* sp. from Direcik I, *B. pikermiensis*, *B. rosamariae*, *B. hellenicus*, and *B. uenayae* (Sarica-Filoreau, 2002; de Bruijn, 1976; López-Antoñanzas et al., 2024; Freudenthal, 1970; Ünay, de Bruijn & Suata-Alpaslan, 2006; Rummel, 1998). Conversely, in *Hispanomys*, M3 always exhibits a distinct and more labially positioned metacone and shows a progressive reduction of the longitudinal crest, which completely disappears in the most derived species such as *Hispanomys adroveri* and *Hispanomys romeroi* (Agustí, 1986; García-Alix et al., 2008; Piñero & Agustí, 2017). Complete loss of the longitudinal crest, however, has never been observed in the M3 of any known species of *Byzantinia*.

Interestingly, as noted above, the single M3 of *Byzantinia* from Zahleh 16 is quite elongated, with a markedly reduced posterior region. The metacone is lingually shifted and so reduced that it is virtually indistinguishable from the posteroloph, whereas the hypocone remains recognizable as a distinct cusp. All of these are typical characters of a derived species of *Byzantinia*. Notably, in this Lebanese specimen, more lingual position of the metacone causes the anterior metaloph (21) to form a spur that is virtually aligned with the long axis of the occlusal surface, a condition that contrasts with the classical, more labially positioned metaloph observed in more primitive species of *Byzantinia*, such as *B. cariensis*, *B. candirensis*, and *B. eskihisarensis*. A similarly lingually displaced anterior metaloph seems to be also present, albeit more vestigial, in the single M3 of *B. pikermiensis* from Çorakyerler (R. López-Antoñanzas and P. Peláez-Campomanes, 2025, personal observations; see also Ünay, de Bruijn & Suata-Alpaslan, 2006, Plate 2, Fig. 28), suggesting a retained ancestral trait.

Lower molars are much more conservative than upper ones, and in fact, it is sometimes difficult to assign lower molars to a particular species of *Byzantinia* based on morphology alone, as they undergo far fewer changes over the course of evolution. In early representatives, such as *Cricetodon pasalariensis* from Pasalar, *B. candirensis* from Çandır, and *B. cariensis* from Sarıçay, the metalophulid on m1 is exclusively or predominantly posterior, representing the plesiomorphic condition within the group. Beginning with *B. sofcaensis* from Sofça and *B. eskisarensis* from Yeni Eskihisar, this configuration shifts toward a double connection, a derived state that characterizes more advanced members of *Byzantinia*. This transition from a single metalophulid II to a doble connection (metalophulid I + metalophulid II) on first lower molars represents a key morphological step in the evolutionary trajectory of derived *Byzantinia* species.

Co-occurrence of two *Byzantinia* species at a given site is not unusual, as this is documented in several Late Miocene localities. These assemblages typically comprise a larger taxon and a smaller one, the latter often exhibiting a funnel-shaped structure (Sen, 2016). Examples of such sites include Direcik I (*Byzantinia* sp. and *B*. aff. *dardanellensis*; Sarica-Filoreau, 2002), Bayraktepe I (*B. bayraktepensis* and *B. ozansoyi*; Ünay, 1980), Bayraktepe II (*B. nikosi* and *B. dardanellensis*; Ünay, 1980), Çorakyerler (*B. hellenicus* and *B. pikermiensis*; Ünay, de Bruijn & Suata-Alpaslan, 2006) and the lower levels of the Zahleh Formation (*B. rosamariae* and *B. dardanellenis* at Zahleh 3; López-Antoñanzas et al., 2024).

Prior to their discovery at the Lebanese locality of Zahleh 16, *Byzantinia pikermiensis* and *B. hellenicus* were known exclusively from Greek (*e.g*., Samos 4 and Pikermi 4; Freudenthal, 1970; Black, Krishtalka & Solounias, 1980) and Anatolian sites (*e.g*., Düzyayla, Hayranlı, Altintaş, and Çorakyerler; de Bruijn, Ünay & Hordijk, 2013; Ünay, de Bruijn & Suata-Alpaslan, 2006). Critically, however, the co-occurrence of these two specific species had been documented only at Çorakyerler in Turkey. Previous biochronological and magnetostratigraphic studies of the Çorakyerler site generally suggested an early–middle Turolian age (*e.g., *Köhler, 1987; Ünay, de Bruijn & Suata-Alpaslan, 2006; Güleç et al., 2007; Geraads, 2013; Kaya et al., 2016). More recent work, however, indicates an older age. Initially considered Vallesian (Becker-Platen, Sickenberg & Tobien, 1975), the fauna was later correlated with MN11, and subsequently proposed to be slightly younger than Pikermi (MN12; Geraads, 2013). Magnetostratigraphic correlations by Kaya et al. (2016) placed the site within chron C4n (8.11–7.64 Ma), although an alternative interpretation aligns it with the C4An–C4r boundary, yielding an older estimated age of ~8.9 Ma. This older “late Vallesian–earliest Turolian” age, which is supported by Kostopoulos et al. (2020, 2021) and Sevim-Erol et al. (2023), appears more consistent with the faunal assemblage. Therefore, the co-occurrence of *B. pikermiensis* and *B. hellenicus* at Zahleh 16 suggests a comparable age and a tentatively biostratigraphic correlation of this level with the MN11 unit. Such an assignment would place Zahleh 16 at least 1 million years younger than the oldest known locality from the upper part of the Zahleh Formation, dated to approximately 10.9–10.0 Ma (López-Antoñanzas et al., 2019, 2024). Nevertheless, a detailed study of the remaining micromammal assemblage of both levels is required to fully confirm this interpretation.

## Conclusions

*Byzantinia* is a cricetodontine rodent that originated in Anatolia and diversified during the late Middle Miocene. This genus is geographically restricted, recorded only from Greece, Turkey, Lebanon, and Romania. Recently, remains have been reported from the lowermost levels (layer 3) of the Lebanese upper Zahleh Formation (*circa* 10.5 Ma). Another locality from the same formation, Zahleh 16, has yielded remains of *B. pikermiensis* and *B. hellenicus*. Here, we provided a description of these fossils. Evolutionary trends within *Byzantinia* include posterior reduction in width of the upper molars, posterior swift of the paracone-paraloph, and loss of the posterior protoloph, resulting in the disappearance of the funnel. In the third upper molars, the metacone tends to be reduced and displaced lingually, becoming hardly distinguishable from the posteroloph. These traits are shared by both *B. pikermiensis* and *B. hellenicus*, which are among the most derived species of the genus. The presence of these two taxa in Zahleh 16 allows us to estimate the locality’s age at approximately 8.9–7.5 Ma (MN11), at least 1 Ma younger than the layer 3 from Zahleh (*circa* 10.9–10 Ma). This makes *Byzantinia* from Zahleh 16 one of the most recent records of the genus, suggesting that it persisted in this region for over 1 million years.

## Supplemental Information

10.7717/peerj.21543/supp-1 Supplemental Information 1 Specimens used in this study.

## Additional Information and Declarations

### Field Study Permissions

The following information was supplied relating to field study approvals (*i.e*., approving body and any reference numbers):

Field work was approved by the Natural Sciences Department of the Lebanese University (Fanar, Lebanon).

### Data Availability

The following information was supplied regarding data availability:

The data is available in the Figures, Tables, and Supplemental File.
