# Supplementary material for: New insights into the genus Byzantinia (Cricetodontinae, Rodentia) from the Late Miocene of Lebanon
Source: PeerJ. 2026 Jul 22;14:e21543. doi: 10.7717/peerj.21543 (PMC13401363; doi:10.7717/peerj.21543)
Supplement: Supplemental Information 1 [file peerj-14-21543-s001.docx]

| **Taxon** | **Locality** | **Institution** | **Material** | **Temporal range** | **References** |
| --- | --- | --- | --- | --- | --- |
| *Byzantinia candirensis* | Çandir (Turkey) | MTA | 263 teeth | 15-13.4 | Tobien, 1978; Rummel, 1998; De Bruijn et al., 2003 |
| *Byzantinia cariensis* | Sariçay (Turkey) | MTA | 49 slides (96-144) >400 teeth | 13.8-11.2 | Sen & Ünay, 1979; Rummel, 1998 |
| *Byzantinia eskihisarensis* | Yeni Eskihisar (Turkey) | MTA | ME-168, ME-252; 348 teeth | 13.8-11.2 | Tobien, 1978; Rummel, 1998 |
| *Byzantinia sofcaensis* | Sofça (Turkey) | MTA | KS-0266, KS-0227, KS-0228, KS-0231, KS-0234, KS-0235, KS-0237, KS-0238, KS-0242, 245, KS-0408, KS-0409, KS-0413,KS-0428, KS-0239, KS-0460, KS-0461, KS-0462,KS-0464, KS-0471 | 13.8-11.2 | Tobien, 1978; Rummel, 1998 |
| *Byzantinia ozansoyi* | Bayraktepe I, Bagici (Turkey) | MTA | >8 and 122 teeth | 13.8-11.2 | Ünay, 1980; Rummel, 1998 |
| *Byzantinia bayraktepensis* | Bayraktepe I, Yeni Eskihisar, Mahmutköy (Turkey) | MTA | >41, 297 and 59 teeth | 13.8-10.7 | Ünay, 1980; Rummel, 1998 |
| *Byzantinia sp, from Direcik I* | Direcik I (Turkey) | MTA | DI 3024, 3030, 3031, 3033, 3034, 3035, 3037, 3043, 3044 | 13.8-11.2 | Sarica-Filoreau, 2002 |
| *Byzantinia rosamariae* | Zahleh 3 (Lebanon) | LUNHM | 12 teeth | 11.5-10.5 | López-Antoñanzas et al.2024 |
| *Byzantinia nikosi* | Biodrak (Greece) | UU | 10 teeth | 9.1-8.9 | De Bruijn 1976 |
| *Byzantinia dardanellensis* | Bayraktepe II (Turkey), Zahleh 3 (Lebanon) | MTA, LUNHM | >40 teeth | 12.2-11 | Ünay, 1980, López-Antoñanzas et al.2024 |
| *Byzantinia pikermiensis* | Pikermi Chomateri (Greece) | UU | 15 teeth | 9.5-7 |  |
| *Byzantinia uenayae* | Karaözü (Turkey) | MTA | 29 teeth | 9.5-8.5 | Rummel, 1998 |
| *Byzantinia hellenicus* | Samos (Greece) | AMNH, UCM, CM | AMNH 20766, UCM 43604, 43605, CM 36258, CM 36269,CM 36270, AMNH 23000, UCM 43615-43618, CM 36260,36261, UCM 43606, 43610, 43619, CM 36266, 36280, UCM 43607, UCM 43620, 43621, 43623, CM 36274-36276, 36290, UCM 43608, 43609,43611,43622,43624,36281, 36289, UCM 43612-43614 and CM 36283 | 8.5-7.2 | Freudenthal, 1970, Black et al. 1980 |

**Abbreviations**

AMNH- American Museum of Natural History

MTA- General Directorate of the Mineral Research & Exploration

UCM- University of Colorado Museum

CM - Carnegie Museum of Natural History

LUNHM- Lebanese University Natural History Museum.

UU-Faculty of Earth Sciences, Utrecht University

Table S1. Specimens used in this study
